# Perioperative hypoalbuminemia predicts postoperative survival: a large cohort study with global context

**DOI:** 10.3389/fmed.2026.1845645

**Published:** 2026-06-30

**Authors:** Yangqi Chu, Shiqian Huang, Pu Chen, Yuxi Zhou, Yu Wang, Xiangdong Chen, Yun Lin

**Affiliations:** 1 Department of Anesthesiology, Union Hospital, Tongji Medical College, Huazhong University of Science and Technology, Wuhan, China; 2 Department of Anesthesiology, Institute of Anesthesia and Critical Care Medicine, Union Hospital, Tongji Medical College, Huazhong University of Science and Technology, Wuhan, China; 3 Key Laboratory of Anesthesiology and Resuscitation (Huazhong University of Science and Technology), Ministry of Education, Wuhan, China

**Keywords:** Global Burden of Disease (GBD), hypoalbuminemia, perioperative management, postoperative mortality, protein-energy malnutrition (PEM)

## Abstract

**Background:**

Protein-energy malnutrition (PEM) remains a global health concern. Although hypoalbuminemia is widely used as a clinically accessible indicator associated with nutritional and inflammatory status, its impact on postoperative survival has not been fully characterized. This study combined global epidemiological analysis with a large-scale clinical cohort to provide complementary insights into the burden of PEM and the prognostic value of perioperative hypoalbuminemia.

**Methods:**

Global trends in PEM-related mortality, prevalence, and disability-adjusted life years (DALYs) from 1990 to 2021 were assessed using Global Burden of Disease (GBD) 2021 data. In parallel, a retrospective cohort of 200,608 surgical patients (2014–2018) from a tertiary hospital was analyzed. Propensity score matching (PSM) and Cox proportional hazards models were applied to evaluate the association between perioperative hypoalbuminemia (serum albumin < 35 g/L) and short-term (3-month) and long-term (3-year) postoperative mortality.

**Results:**

GBD 2021 analysis showed a decline in PEM-related mortality and DALYs from 1990 to 2021, while prevalence decreased more slowly. In the clinical cohort, perioperative hypoalbuminemia was associated with increased postoperative mortality. After PSM, preoperative hypoalbuminemia was associated with higher short-term (HR 2.45, 95% CI 2.16-2.77) and long-term mortality (HR 1.63, 95% CI 1.55–1.72), while postoperative hypoalbuminemia showed stronger associations (HR 3.15 and 1.73, respectively). Associations were consistently observed across sex- and age-specific subgroups.

**Conclusions:**

This study highlights the continuing burden of PEM and shows that hypoalbuminemia is associated with poor postoperative outcomes. The stronger association of postoperative hypoalbuminemia with short-term mortality suggests that postoperative nutritional assessment may warrant further prospective evaluation.

## Background

Protein-energy malnutrition (PEM) refers to a nutritional deficiency resulting from inadequate intake of protein and/or energy, or an imbalanced intake that impairs the body's ability to maintain normal physiological functions (1–3). PEM is widely recognized as a significant adverse prognostic factor in various clinical conditions, with the most severe forms manifesting as marasmus, kwashiorkor, and marasmic kwashiorkor (1, 4, 5). Despite progress in public health and healthcare systems, which has led to a reduction in the overall burden of PEM, including mortality, its high global prevalence remains a persistent challenge to global health (2, 6, 7). Given this, a deeper understanding of the global burden of PEM and its evolving trends is crucial for guiding the formulation of effective intervention strategies tailored to the specific characteristics of different regions and populations.

Despite the growing concern regarding PEM, research on its global burden remains relatively limited. Studies indicate that the Nordic region has the lowest prevalence of malnutrition, whereas Southeast Asia exhibits the highest prevalence worldwide (8). Furthermore, regions with lower socio-demographic indices tend to have a lower age-standardized rate of these nutritional deficiencies, which may be closely linked to socioeconomic factors, food insecurity, and unequal access to healthcare resources (7). Collectively, these factors exacerbate the risks associated with PEM, particularly in low- and middle-income countries.

Albumin (ALB) is a plasma protein synthesized by the liver, which plays an important role in maintaining blood osmotic pressure, transporting various substances, and immune regulation (9). Hypoalbuminemia has traditionally been regarded as a clinically relevant surrogate indicator of PEM, reflecting impaired nutritional reserve and metabolic dysfunction in the body (2, 10, 11). However, serum ALB is a multifactorial biomarker whose circulating concentration is influenced not only by nutritional status but also by systemic inflammation, liver function, fluid balance, perioperative physiological stress, and other non-nutritional factors (12, 13). Therefore, in the perioperative setting, hypoalbuminemia should not be interpreted as a PEM-specific diagnostic biomarker, but rather as a multifactorial marker reflecting nutritional status, systemic inflammation, and overall physiological condition. Consistent with this perspective, current perioperative nutrition guidelines from the European Society for Clinical Nutrition and Metabolism emphasize routine nutritional risk screening and early nutritional optimization as essential components of perioperative care (14, 15). In parallel, existing clinical studies conducted in hospital settings have observed that patients with hypoalbuminemia tend to experience worse perioperative outcomes. Multiple studies have demonstrated that perioperative hypoalbuminemia is closely associated with increased postoperative mortality, delayed recovery of quality of life, and higher risks of surgical site infections and thromboembolic events (16–19). Nevertheless, most hospital-based studies are limited by their focus on specific types of surgeries or isolated time points (such as preoperative or postoperative ALB levels), and fail to comprehensively elucidate how hypoalbuminemia impacts both short- and long-term survival outcomes.

To bridge the gap between population-level nutritional burden and real-world perioperative prognosis, this study integrated GBD 2021 data with perioperative data from a large tertiary hospital in China, with the GBD analysis providing a global epidemiological context and the clinical cohort examining the association between perioperative ALB levels and postoperative survival. The findings may provide evidence to support perioperative risk assessment.

## Methods

### Study design

This study comprised two components: a global epidemiological analysis based on the GBD 2021 database and a retrospective cohort study conducted at a tertiary hospital to evaluate the association between perioperative hypoalbuminemia and postoperative survival. The cohort analysis was the primary component, while the GBD analysis provided global epidemiological context.

### GBD 2021 data analysis

The GBD 2021 provides a comprehensive assessment of 204 countries and regions, spanning 371 diseases, 88 risk factors, and 288 causes of death from 1990 to 2021 (20). Data are stratified by age and sex, enabling detailed analyses. Detailed methodologies, including data sources, strategies for data quality improvement, and statistical models, are available in the literature (21, 22).

PEM-related data were extracted from the GBD 2021 database using the disease category “protein-energy malnutrition,” as defined by the GBD cause hierarchy (23, 24). The burden of PEM was evaluated across three dimensions: prevalence, mortality, and DALYs, which reflect the extent of PEM's prevalence, its lethality, and its overall impact on quality of life. DALYs were determined by summing the years of life lost (YLL) due to premature death and the years lived with disability (YLD), providing a comprehensive measure of disease burden.

### PEM data collection and analysis

Global PEM burden data from 1990 to 2021 were retrieved using the GBD Results Tool. Longitudinal trends in mortality, DALYs, and prevalence were analyzed through comparisons between 1990 and 2021, adjusted for sex, age, and age-standardization. For predefined calendar periods (1990–1999, 2000–2009, 2010–2021, and the full period of 1990–2021), average annual percent change (AAPC) was calculated using log-linear Joinpoint regression with 0 joinpoints to assess temporal trends and their statistical significance.

Subgroup analyses by sex, age and region were conducted to identify high-risk populations and distribution differences, which may help inform future targeted prevention and management strategies. Additionally, contemporary PEM epidemiology was assessed using 2021 cross-sectional data, comparing mortality, DALYs, and prevalence across sex and age groups. These findings may provide epidemiological evidence relevant to public health planning.

### Collation and analysis of perioperative data

Building on the GBD studies, this study assessed the association between perioperative hypoalbuminemia and postoperative mortality. Conducted at Wuhan Union Hospital, Hubei, China, a tertiary comprehensive center, this retrospective cohort study adhered to the STROBE reporting guideline and was ethically approved (20210641) on July 19, 2023, with no requirement for informed consent due to the use of non-personally identifiable aggregated data. The study included patients undergoing surgery and anesthesia from January 2014 to December 2018. Patients without available perioperative ALB measurements within the predefined time window were excluded from the corresponding analyses, as detailed in eMethod 1 in Supplementary File S1.

Hypoalbuminemia was defined as serum ALB levels < 35 g/L, with data collected on pre- and postoperative ALB levels. Preoperative albumin was defined as the most recent measurement within 7 days before surgery. Postoperative ALB was measured within 7 days after surgery. The primary outcome was postoperative mortality, ascertained through deterministic linkage with China's National Disease Surveillance Points (DSP) system, a nationally validated mortality surveillance system with regular quality-control procedures, covering the period from 2014 to 2021, and categorized as short-term (3-month) and long-term (3-year) postoperative mortality (eMethod 1 in Supplementary File S1).

To control for confounding factors, propensity score matching (PSM) was employed, incorporating patient-related, surgical, and anesthesia-related variables. The confounding variables included in this study were (1) patient-related factors: sex, age, body mass index (BMI), American Society of Anesthesiologists physical status (ASAPS); previous surgical history, previous heart disease history, previous diabetes history, previous hypertension history, previous cerebral infarction history, history of drinking, history of smoking, preoperative antihypertensive medication use, preoperative corticosteroid medication use; (2) surgical-related factors: duration of surgery, type of surgery (categorized according to the primary surgical specialty recorded in the hospital electronic medical system, including ear, nose & throat [ENT], Gynecological, Gastrointestinal tract, Orthopedic, Urological, General, Nervous system, Cardiac, Thoracic, and Ophthalmic surgery), surgery grade, emergency surgery, night shifts; (3) anesthesia-related factors: type of anesthesia, combined intravenous and inhalation anesthesia, transfusion; intraoperative blood transfusion amount, intraoperative use of vasoactive drugs. The specific definitions of the matching variables are outlined in eMethod 1 in Supplementary File S1.

Statistical analysis included descriptive statistics for clinical data, preliminary assessments using *t*-tests and chi-squared tests. A multivariable logistic regression model was used to estimate the propensity scores. The 1:1 matching protocol without replacement, along with a caliper of 0.05 SD of the logit of the propensity score, was employed. Standardized mean differences (SMDs) were computed for the PSM cohorts to assess baseline balance following PSM. After 1:1 PSM using the MatchIt package (R), Cox proportional hazards regression stratified by matched pair [strata(pair_id)] was performed to compare survival between patients with and without hypoalbuminemia. Robust sandwich variance estimators with clustering by matched pair [cluster(pair_id); robust = TRUE] were used to account for within-pair correlation and to obtain valid hazard ratios (HRs) and 95% confidence intervals (CIs) for long- and short-term postoperative mortality. Variables with >10% missingness were excluded from the propensity score model, while variables with ≤ 10% missingness were imputed using multiple imputation (MICE, 5 imputations). Specifically, socioeconomic factors with >10% missingness, including annual household income (14% missing) and highest educational level (12% missing), were excluded. BMI had 9,725 missing values (4.85%), and intraoperative blood transfusion amount had 4,890 missing values (2.43%); both were imputed. We also performed a sensitivity analysis comparing results from the multiple imputation approach with those from complete-case analysis (excluding any patient with missing covariates). All analyses were performed using R version 4.2.0, with “MatchIt” and “survival” packages for PSM and regression analyses, respectively (eMethod 2 in Supplementary File S1). All data analyses, including those based on GBD 2021 data and retrospective clinical data, were performed in accordance with the relevant guidelines and regulations.

## Results

## Overall assessment of the global burden of PEM

Globally, in 2021, there was a downward trend in both deaths and DALYs attributed to PEM when compared to 1990. As illustrated in Table 1, which compares PEM-related mortality, DALYs, and prevalence between 1990 and 2021, both the absolute numbers and age-standardized rates of mortality and DALYs related to PEM experienced a significant decline. However, it is important to note that the prevalence of PEM has decreased at a much slower rate than mortality and DALYs, and even by 2021, the number of people affected by PEM surpassed the levels observed in 1990. Specifically, the global prevalence of PEM increased from 113.02 million in 1990 (95% CI: 104.58 million to 123.62 million) to 127.22 million in 2021 (95% CI: 115.16 million to 142.91 million). This trend suggests that, despite a reduction in PEM's overall burden, the prevalence continues to rise, highlighting that PEM remains a persistent challenge with a substantial impact on quality of life.

**Table 1:** Global trends in mortality, DALYs, and prevalence of protein-energy malnutrition from 1990 to 2021.

Indicator	1990	2021	Changes (%)
DEATH	
Numbers^b^	505,632.39(429,698.14–619,494.57)	189,345.50(167,550.85–213,754.71)	−62.55
Rate^a, b^	9.48(8.06–11.61)	2.40(2.12–2.71)	−74.68
Age-standardized rate^b^	9.44(8.16–11.32)	2.61(2.29–2.98)	−72.35
**DALYs** ^c^	
Numbers^b^	42,012,720.56(35,549,209.61–52,508,991.25)	11,826,130.80(9,881,913.81-13,963,539.45)	−71.85
Rate^a, b^	787.70(666.51–984.49)	149.86(125.22–176.95)	−80.97
Age-standardized rate^b^	699.30(594.81–869.67)	172.38(142.75–204.10)	−75.35
**PREVALENCE**	
Numbers (in millions)^b^	113.02(104.58–123.62)	127.22(115.16–142.91)	12.56
Rate^a, b^	2,118.95(1,960.69–2,317.67)	1,612.12(1,459.33–1,810.98)	−23.92
Age-standardized rate^b^	1,986.52(1,827.52–2,186.62)	1,696.86(1,548.51–1,892.08)	−14.58

a “Rate” is expressed per 100,000 population, and “Age-standardized rate” is adjusted to the global standard population.

b Estimates are presented with 95% confidence intervals in parentheses.

c DALYs, disability-adjusted life-years.

The age-standardized AAPC values for mortality, DALYs, and prevalence rates of PEM from 1990 to 2021 are presented in Table 2. Over the past three decades, the AAPC values for these three indicators have exhibited an overall downward trend, with mortality and DALYs showing a marked reduction. During the predefined periods of 2000–2009 and 2010–2021, mortality and DALYs continued to decrease significantly (*P*-values < 0.001). In contrast, the prevalence rate showed a different temporal pattern, with a slight increase during 1990–1999, followed by significant declines after 2000. Despite the overall downward trend, the prevalence rate in 2021 remained higher than that in 1990, consistent with the findings presented in Table 1.

**Table 2:** Average annual percent change of mortality, DALYs, and prevalence rates of Protein-Energy Malnutrition (1990–2021).

**Time period**	**Death rate** ^**a**^		**DALYs rate** ^**a, c**^		**Prevalence rate** ^**a**^	
	**AAPC (95% CI)** ^c^	***P*** **value**	**AAPC (95% CI)** ^c^	***P*** **value**	**AAPC (95% CI)** ^c^	***P*** **value**
1990–1999	−0.255 (−1.964, 1.427)	0.723	−0.677 (−2.073, 0.678)	0.292	0.449 (0.427, 0.470)	**< 0.001** ^b^
2000–2009	−7.411 (−8.922, −5.875)	**< 0.001** ^b^	−6.877 (−8.132, −5.605)	**< 0.001** ^b^	−0.084 (−0.123, −0.044)	**0.001** ^b^
2010–2021	−6.288 (−8.743, −3.980)	**< 0.001** ^b^	−7.485 (−9.505, −5.568)	**< 0.001** ^b^	−2.318 (−3.301, −1.324)	**< 0.001** ^b^
1990–2021	−4.466 (−4.962, −3.966)	**< 0.001** ^b^	−4.637 (−5.126, −4.146)	**< 0.001** ^b^	−0.280 (−0.513, −0.047)	**0.020** ^b^

a “Rate” is expressed per 100,000 population.

b Bold values indicate statistical significance (*P* < 0.05).

c CI, confidence interval; AAPC, Average Annual Percentage Change; DALYs, Disability-Adjusted Life Years.

**Figure 1:**
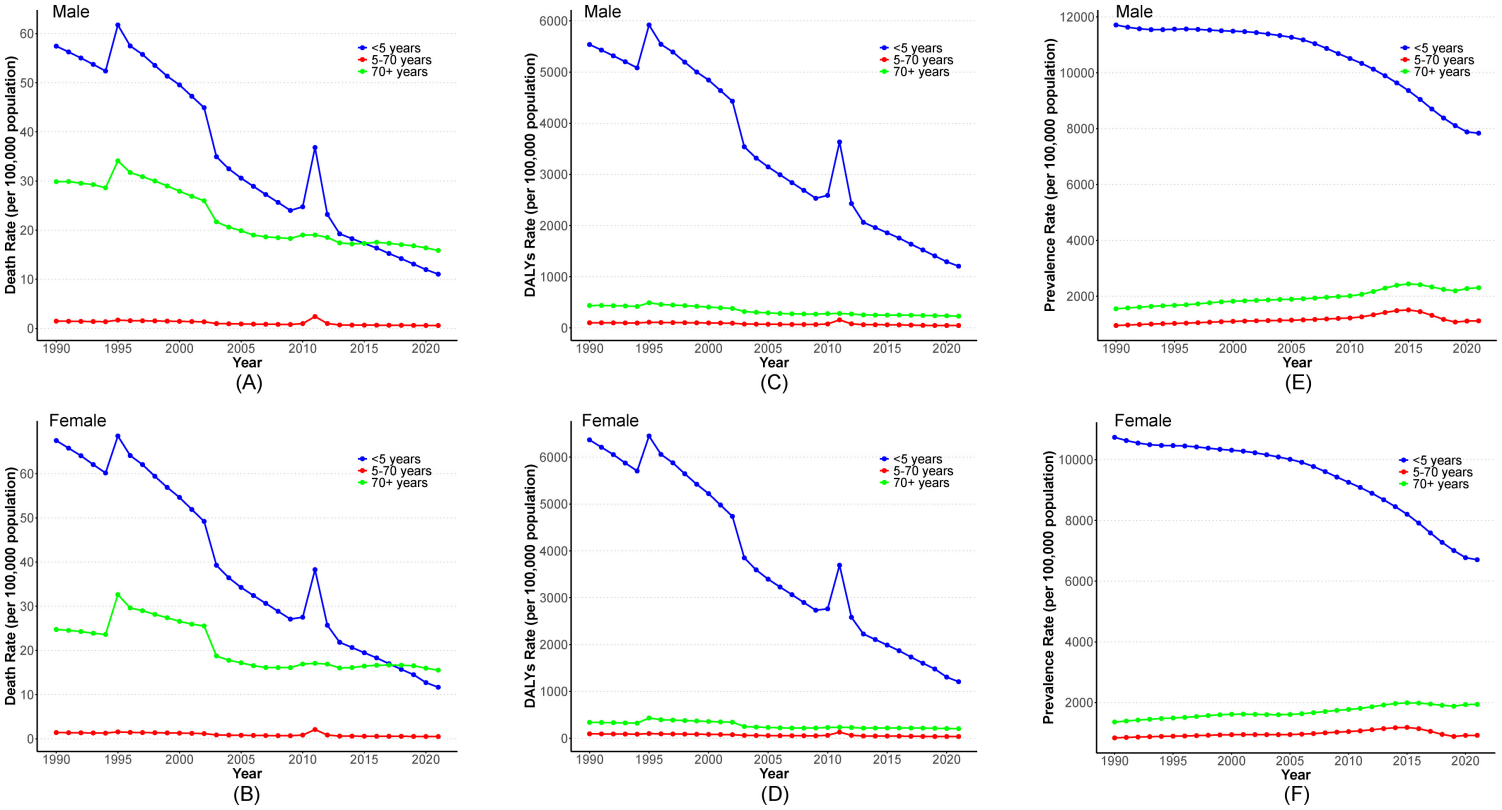
Trends in PEM-related mortality, DALYs, and prevalence rates by age and sex, 1990–2021. This figure illustrates the trends in PEM burden for different age groups (< 5 years, 5–70 years, >70 years) and genders from 1990 to 2021. The top row shows male data and the bottom row shows female data.

**Figure 2:**
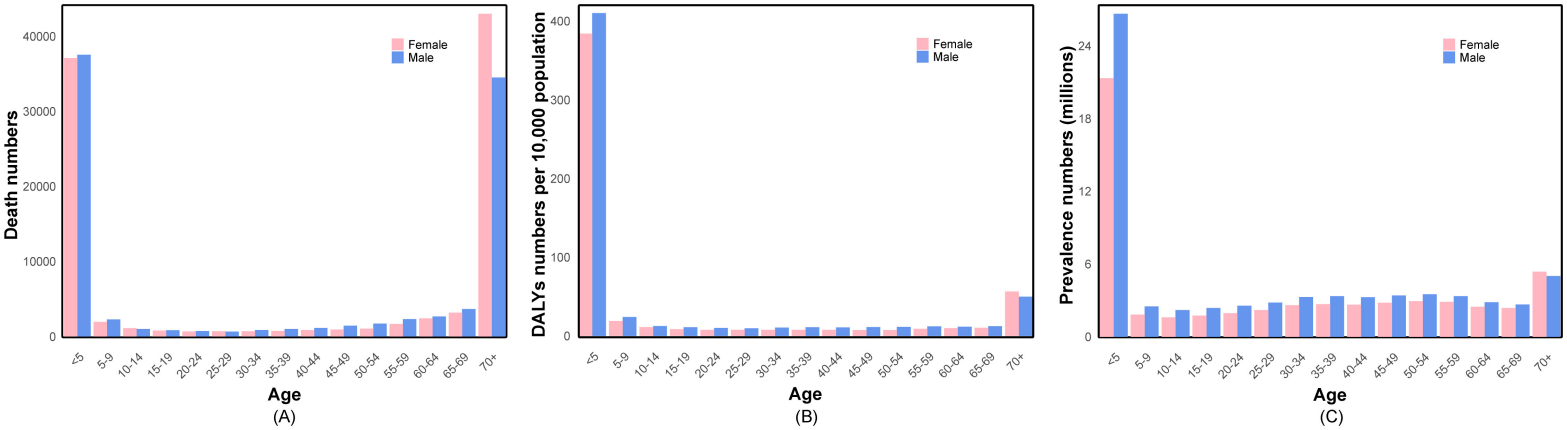
Age and sex distribution of PEM-related mortality, DALYs, and prevalence in 2021. Age and sex distribution of PEM-related deaths **(A)**, DALYs **(B)**, and prevalence **(C)** in 2021. Pink bars represent females and blue bars represent males.

Age differences indeed significantly influence the occurrence of PEM. In both males and females, the burden of PEM is most pronounced in children under 5 years of age, while the impact on the adult population aged 5–70 years is relatively milder. As depicted in Figure 1, the mortality risk associated with PEM is notably higher in individuals over 70 years old. Alarmingly, this trend has not been effectively mitigated over the past few decades, with no clear indication of a decline in mortality rates. Regarding the prevalence of PEM, although there has been a slight decrease in the prevalence among children under 5 in recent years, the rate of decline has been too slow, and the overall incidence remains inadequately controlled. Furthermore, the prevalence of PEM is rising in both the adult population aged 5–70 years and the elderly population over 70. Across genders, the trends in PEM occurrence are generally similar, with males exhibiting a slightly higher prevalence than females. The most recent 2021 data reveal that the PEM burden remains higher in the < 5 years and >70 years age groups, with no significant gender differences observed (Figure 2).

## The impact of regional differences on PEM burden

**Figure 3:**
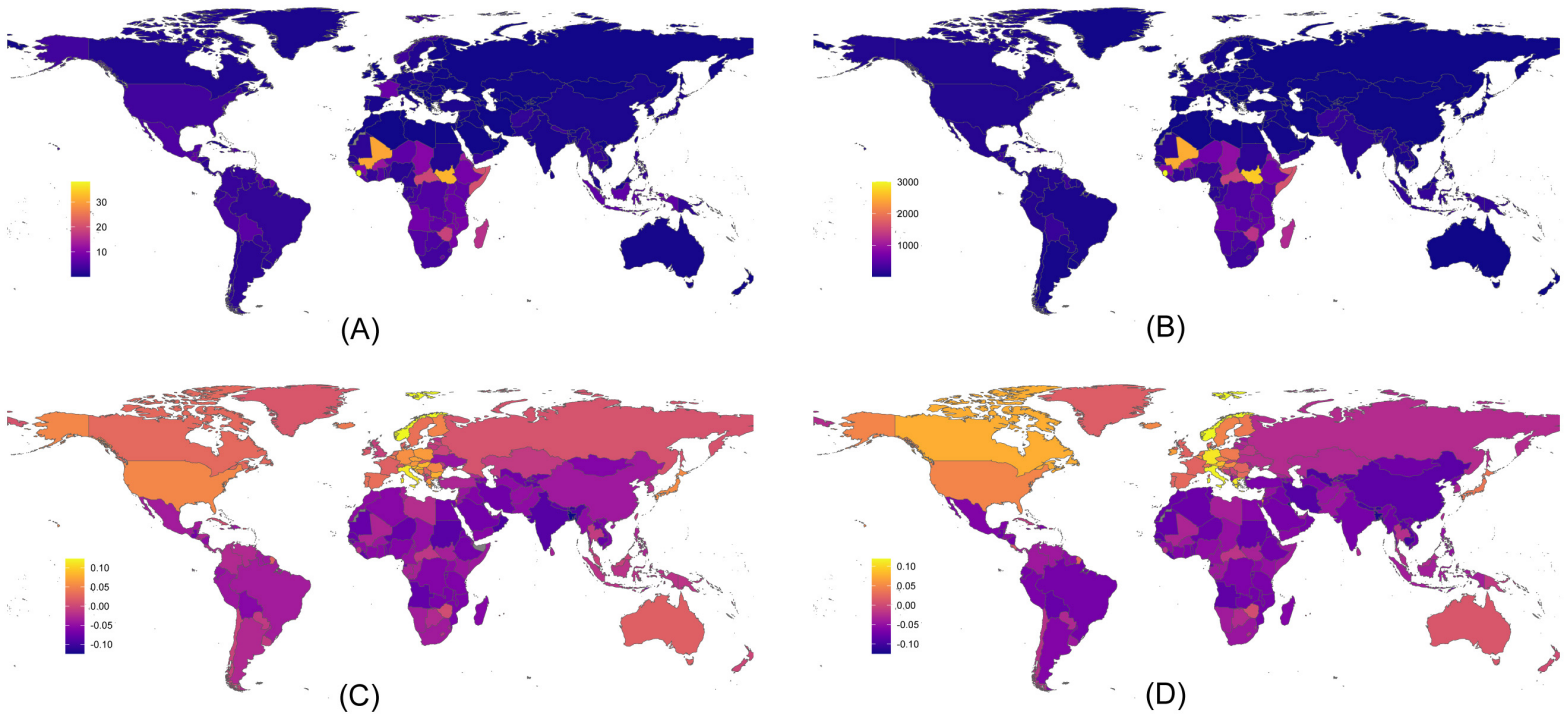
Regional distribution and trends of PEM burden. **(A)** The regional distribution of PEM mortality rate in 2021. **(B)** The regional distribution of PEM DALYs rate in 2021. **(C)** The change in PEM mortality rate from 1990 to 2021. **(D)** The change in PEM DALYs rate from 1990 to 2021.

Across the 204 GBD countries, in 2021, the countries with the highest PEM-related mortality rates were Sierra Leone (38.17, 95% CI: 25.28–55.31), South Sudan (31.97, 95% CI: 21.33–47.73), and Mali (30.55, 95% CI: 20.37–42.51). These countries also exhibited the highest DALYs rates, with Sierra Leone at 3,006.46, South Sudan at 2,678.46, and Mali at 2,452.74. Conversely, the countries with the lowest mortality rates were Kuwait (0.0053, 95% CI: 0.0044–0.0063), Andorra (0.023, 95% CI: 0.014–0.033), and Georgia (0.037, 95% CI: 0.029–0.042). Similarly, the countries with the lowest DALYs rates were Kuwait, Georgia, and Croatia (Figure 3).

Between 1990 and 2021, the countries with the highest percentage increase in mortality rates were Norway (12.38%), Italy (11.57%), and Croatia (9.33%), while Bangladesh (−0.12%), Bhutan (−0.11%), and the Maldives (−0.09%) had the lowest growth rates. Regarding DALYs rates, the highest growth was observed in Norway (0.12%), Italy (0.12%), and Andorra (0.11%), while the countries with the lowest growth rates were Kuwait (−0.12%), Bangladesh (−0.12%), and the Maldives (−0.11%) (Figure 3).

## Postoperative survival analysis of hypoalbuminemia

A total of 200,608 patients undergoing surgery at a tertiary hospital between 2014 and 2018 were included in the retrospective cohort analysis. Tables S1 and S2 (Supplementary File S1) provide insights into the baseline characteristics and survival data of the surgical patients. On average, patients with hypoalbuminemia were older than those without hypoalbuminemia, both preoperatively (46.9 vs. 38.9 years) and postoperatively (44.5 vs. 34.1 years). Additionally, the hypoalbuminemia group had a higher proportion of women compared to the non-hypoalbuminemia group (preoperative group: 52.1% vs. 48.2%; postoperative group: 49.8% vs. 47.4%). Patients with hypoalbuminemia were also more likely to have higher ASA scores, and a higher prevalence of preoperative comorbidities, such as a history of prior surgery, hypertension, diabetes, smoking, alcohol consumption, or the use of glucocorticoids or antihypertensive medications. Notably, patients with hypoalbuminemia experienced a higher incidence of night-time surgeries and emergency procedures.

PSM was conducted at a 1:1 ratio for the entire cohort and stratified cohorts, including male, female, age > 70 years, age < 5 years, and age 5–70 years cohorts. After the PSM, the distribution of confounders between the groups was relatively balanced in age > 70, age < 5, age between 5 and 70 years, male, female cohort and the entire cohort, with the SMD distributions depicted in Figure S1~6 (Supplementary File S1). For the analysis of preoperative hypoalbuminemia, the number of deaths within 3 years / 3 months in each subgroup was: entire cohort, 5,451 / 862; age >70 years, 1,065 / 232; age < 5 years, 22 / 10; age between 5 and 70 years, 4,028 / 941; male, 3,582 / 803; female, 1,890 / 409. For the analysis of postoperative hypoalbuminemia, the event numbers were: entire cohort, 5,422 / 1,200; age >70 years, 527 / 75; age < 5 years, 30 / 17; age between 5 and 70 years, 5,243 / 776; male, 3,324 / 476; female, 2,074 / 303.

**Figure 4:**
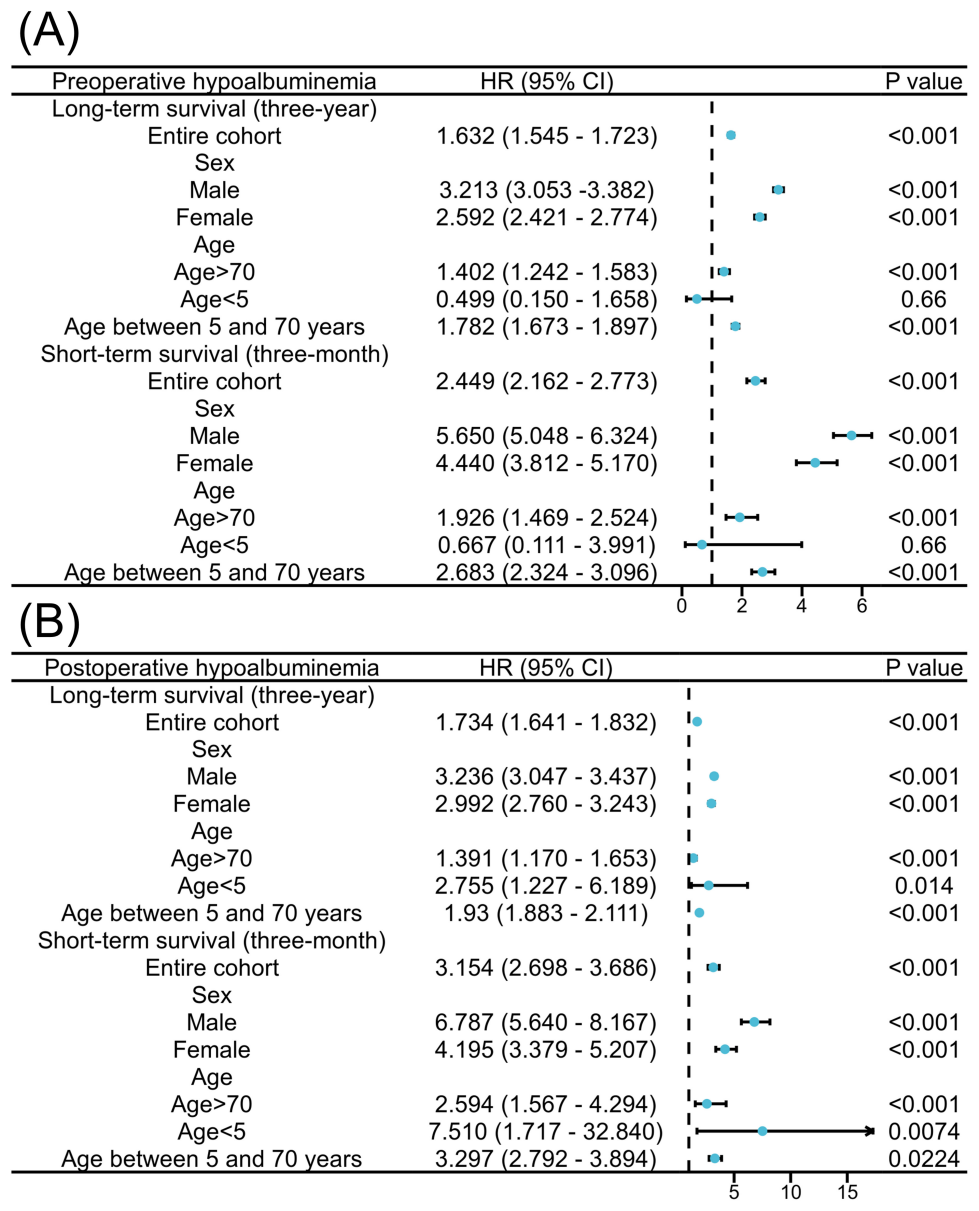
Cox regression analysis and subgroup analysis for long-term survival (3-year) and short-term survival (3-month) and hypoalbuminemia after propensity score matching. **(A)** Cox regression analysis and subgroup analysis for long-term/short-term survival and preoperative hypoalbuminemia. Subgroup sample sizes: entire cohort, *N* = 58,956; Age >70 years, *N* = 5,082; Age < 5 years, *N* = 1,880; Age between 5 and 70 years, *N* = 51,676; Male, *N* = 28,170; Female, *N* = 29,976. **(B)** Cox regression analysis and subgroup analysis for long-term/short-term survival and postoperative hypoalbuminemia. Subgroup sample sizes: entire cohort, *N* = 145,324; Age >70 years, *N* = 3,644; Age < 5 years, *N* = 9,202; Age between 5 and 70 years, *N* = 137,626; Male, *N* = 74,032; Female, *N* = 70,318.

Figure 4 presents the results obtained after PSM and Cox survival regression analysis, evaluating the impact of preoperative and postoperative hypoalbuminemia on short-term (3-month postoperative) and long-term (3-year postoperative) survival. For short-term survival, the HR was 2.449 (95% CI: 2.162, 2.773) in the preoperative hypoalbuminemia population and 3.154 (95% CI: 2.698, 3.686) in the postoperative hypoalbuminemia population. For long-term survival, the HR was 1.632 (95% CI: 1.545, 1.723) in the preoperative hypoalbuminemia population and 1.734 (95% CI: 1.641, 1.832) in the postoperative hypoalbuminemia population. Preoperative hypoalbuminemia was associated with a 2.45-fold higher hazard of short-term mortality and a 1.63-fold higher hazard of long-term mortality compared with patients without hypoalbuminemia. Postoperative hypoalbuminemia was associated with even higher mortality hazards. Kaplan-Meier survival curves for the matched cohorts (Figure S7) consistently showed significantly lower survival probabilities in patients with hypoalbuminemia (both preoperative and postoperative) compared with those without hypoalbuminemia, for both 3-month and 3-year follow-up periods (log-rank *P* < 0.0001 for all comparisons).

To assess the robustness of our findings to missing data handling, we repeated the analysis in a complete-case cohort excluding any patient with missing covariate data. The results were consistent with the primary analysis. For short-term survival, the HR was 2.319 (95% CI: 2.047, 2.628) in the preoperative hypoalbuminemia population and 3.165 (95% CI: 2.684, 3.733) in the postoperative hypoalbuminemia population. For long-term survival, the HR was 1.601 (95% CI: 1.514, 1.693) in the preoperative hypoalbuminemia population and 1.894 (95% CI: 1.785, 2.011) in the postoperative hypoalbuminemia population (Table S3). These HRs are similar to those from the primary imputed analysis, confirming the robustness of our conclusions. Stratified and interaction analysis (Table S4) showed that postoperative hypoalbuminemia was associated with increased mortality across sex-specific subgroups, with HRs numerically higher in males than females (*P* = 0.005 for long-term mortality, *P* = 0.043 for short-term mortality). The hypoalbuminemia-mortality association was also modified by age for patients >70 years (*P* for interaction < 0.05 for long-term and short-term mortality), but not for children < 5 years (all *P* for interaction >0.05), suggesting no statistically significant interaction in the pediatric subgroup. However, given the relatively small sample size in the preoperative cohort (*N* = 1,880), these estimates for children under 5 years of age should be interpreted with caution.

## Discussion

This study is among the first to integrate global epidemiological data with a large-scale clinical cohort to assess the burden of PEM and its impact on perioperative outcomes. In this study, perioperative hypoalbuminemia was strongly associated with increased postoperative mortality, with postoperative hypoalbuminemia showing a more pronounced effect on short-term survival than preoperative hypoalbuminemia. These findings suggest that postoperative ALB decline may primarily reflect underlying disease severity. Postoperative hypoalbuminemia may also serve as a clinically accessible marker for perioperative risk stratification, rather than a directly modifiable therapeutic target. Whether albumin-targeted interventions can directly improve postoperative outcomes remains to be established.

In addition, GBD 2021 analysis showed that although PEM-related mortality and DALYs have declined over the past three decades, the prevalence remains substantial and decreases at a slower rate, indicating a persistent global health burden. This trend may be attributed to several key factors, including improvements in the global economy, advancements in poverty alleviation efforts, and enhancements in literacy and education. Globally, malnutrition and limited access to healthy diets persist, with more than 2.8 billion people, representing over one-third of the global population, unable to afford a healthy diet in 2022 (25). Even among those without financial constraints, poor dietary habits and the appeal of non-nutritive foods contribute to suboptimal nutrition (26, 27). Food insecurity, driven by economic conflicts, climate change, COVID-19, and rising energy costs, further exacerbates the problem by limiting food quality and stability (28, 29).

In terms of age disparities, data from the GBD database suggest that the prevalence of PEM is predominantly observed in two age groups: children under 5 years old and individuals over 70 years old, highlighting the need for continued attention to these vulnerable age groups. In children, PEM is often linked to inadequate protein intake resulting from poor dietary patterns, food allergies, or chronic gastrointestinal disorders (30). This nutritional deficiency is associated with impaired growth, higher susceptibility to infections, and delayed recovery from illness, which may contribute to sustained PEM prevalence (31, 32). GBD data from 1990 to 2021 showed that PEM-related mortality remained consistently high in individuals aged >70 years. In stark contrast to children under 5 years old, the PEM mortality rate in the elderly has remained persistently high throughout the study period. For the elderly, PEM is associated with physiological and pathological changes, including poor appetite, oral health issues, diminished digestive capacity, and sarcopenia, which collectively exacerbate amino acid deficiencies and metabolic imbalances (33). In individuals with severe PEM, immune function tends to be compromised, potentially increasing vulnerability to infections and further affecting overall health status. Age-stratified analysis of hospital data revealed differences compared to GBD findings. These discrepancies may stem from the hospital-based population being limited to surgical patients, who typically receive higher levels of medical intervention. Furthermore, infants and elderly individuals with severe conditions who cannot tolerate surgery are often excluded, potentially underestimating their true burden in hospital data. Differences in data sources, outcome definitions, and patient selection criteria may also contribute to the observed variations.

Unlike previous studies, both hospital and GBD datasets suggest a higher prevalence of PEM in males compared to females; however, although interaction analyses were performed, these subgroup findings should be interpreted as exploratory and cautiously interpreted. This disparity may be related to males' greater protein and energy requirements and higher susceptibility to infectious diseases (23, 34). Hormonal and metabolic differences may also contribute to sex-specific vulnerability (35–37). Previous studies have suggested that males and females may exhibit different inflammatory and immune responses during physiological stress (38, 39). In addition, sex hormone regulation during stress may contribute to differences in immune and metabolic responses, which may subsequently influence perioperative ALB dynamics (40). Therefore, sex-specific inflammatory and hormonal differences may partly explain the observed sex disparities in perioperative hypoalbuminemia. However, these potential mechanisms could not be directly evaluated in the present study and require further investigation.

At the regional level, Sierra Leone, South Sudan, and Mali are among the lowest-income countries with the highest levels of poverty globally. Poverty not only limits people's access to adequate nutrition but also weakens the food supply chain, making it vulnerable to external shocks. Data indicate that, in Sub-Saharan Africa, factors such as drought, violent conflict, and locust infestations are major sources of food crises and malnutrition (41). Sierra Leone and South Sudan have been in a prolonged state of political instability and armed conflict, where malnutrition often becomes a consequence of economic instability and social unrest. Moreover, these countries are also burdened by infectious diseases such as malaria, tuberculosis, and diarrheal diseases, which further exacerbate the burden of PEM. In contrast, Norway and Italy are the countries with the largest increases in the burden of PEM. The rise in this burden may be closely related to population aging. As mentioned previously, the elderly are already considered a high-risk group for PEM. Older adults often face multiple chronic conditions (such as diabetes and heart disease), increased medication use, and a decline in interest in food. These factors collectively contribute to a higher risk of malnutrition (42–44). Additionally, poor dietary habits, such as high consumption of sugar and fat, further increase the risk of developing PEM.

In clinical practice, hypoalbuminemia is often influenced by multiple factors, including inflammation, fluid status, and liver function. Although it has traditionally been used as a clinically relevant surrogate marker of PEM, accumulating evidence suggests that hypoalbuminemia and PEM are also closely associated with impaired quality of life and adverse postoperative outcomes (45, 46). The hospital data analysis in this study demonstrates that hypoalbuminemia is significantly associated with an increased risk of postoperative mortality, both when present preoperatively and postoperatively. Notably, postoperative hypoalbuminemia is associated with a greater reduction in short-term survival. These findings support the potential value of serum ALB monitoring for perioperative risk assessment. Perioperative nutritional assessment and serum ALB monitoring may help identify high-risk patients. Given the stronger association between postoperative hypoalbuminemia and short-term mortality, postoperative ALB monitoring and early postoperative nutritional assessment may be particularly important.

To date, research on the global burden of PEM remains limited, with much of the existing literature relying on outdated data and methodologies. Similarly, most studies addressing hypoalbuminemia in the perioperative setting focus on specific types of surgeries, with few investigating the combined impact of both preoperative and postoperative hypoalbuminemia on patient outcomes. This study is among the first to combine GBD 2021 data with single-center hospital clinical data to examine the global burden of PEM and its potential relevance to perioperative management. The GBD analysis provides a global epidemiological context, while the hospital-based cohort focuses on clinical associations between perioperative hypoalbuminemia and postoperative outcomes. The GBD and hospital-based analyses addressed different but complementary research questions, providing both population-level and clinical perspectives. Key findings include the identification of high-risk populations, such as children under 5 years, the elderly, and males, as well as distinct regional variations in the PEM burden. These findings may support perioperative risk stratification, although prospective validation remains necessary.

Despite its strengths, this study has several inherent limitations. First, the GBD database relies on estimates and statistical models, which can introduce errors, especially in regions with limited data, and its update cycle may not capture the most recent health trends or the impact of emerging public health events. Additionally, serum ALB cannot fully capture the complexity of PEM, thereby limiting the comprehensiveness of nutritional assessments (47, 48). Therefore, the observed association between perioperative hypoalbuminemia and postoperative mortality in this study should be interpreted primarily as a prognostic association rather than evidence of a direct causal or therapeutic relationship. Although PSM and multivariable Cox regression were used to reduce baseline imbalance and control for measured confounders, residual confounding cannot be completely excluded. Important unmeasured variables may include cancer stage, active infection/sepsis, baseline frailty, disease severity, inflammatory status, postoperative complications, and socioeconomic factors. Residual confounding from these factors cannot be completely excluded. Socioeconomic factors are especially relevant to the GBD analysis, as cross-country differences may affect PEM prevalence and outcomes. Many of these factors may influence both serum ALB levels and postoperative mortality, and were not fully captured in the available covariates. Additionally, certain socioeconomic variables, including annual household income and educational level, were excluded from the propensity score model because of missingness >10%, which may have contributed to residual confounding. In particular, postoperative hypoalbuminemia may, in some cases, reflect underlying complications or acute postoperative complications rather than directly contributing to mortality, introducing the possibility of reverse causation. Lastly, hospital data, despite providing detailed clinical information, is restricted to patients who seek medical care and may not fully capture the burden of PEM in high-risk groups who do not access healthcare, leading to a potential underestimation of the global burden of PEM.

Future research should prioritize several key areas. First, in-depth investigations into the nutritional status and physiological differences of high-risk populations are needed to clarify the pathological mechanisms and effects of hypoalbuminemia. Additionally, high-quality randomized controlled trials (RCTs) are essential for evaluating the association between nutritional strategies and postoperative outcomes. For instance, future prospective studies evaluating strategies such as preoperative ALB supplementation may help clarify their potential association with postoperative outcomes. However, as the present study did not evaluate interventional efficacy, whether such approaches provide clinical benefit requires further validation in prospective randomized trials.

## Conclusions

This study combined global epidemiological analysis with a large clinical cohort to evaluate the burden of PEM and its impact on perioperative outcomes. Despite declines in PEM-related mortality and DALYs, its prevalence remains substantial, indicating a persistent global health challenge. Perioperative hypoalbuminemia was associated with reduced postoperative survival, with postoperative hypoalbuminemia showing a stronger effect. These findings suggest that serum ALB may serve as a practical marker for perioperative risk stratification, while the potential clinical value of targeted perioperative nutritional assessment strategies in high-risk patients requires further validation in prospective randomized trials.

## Data Availability

The original contributions presented in the study are included in the article/Supplementary material, further inquiries can be directed to the corresponding authors.
