# Supplementary material for: Perioperative hypoalbuminemia predicts postoperative survival: a large cohort study with global context
Source: Front Med (Lausanne). 2026 Jun 30;13:1845645. doi: 10.3389/fmed.2026.1845645 (PMC13365050; doi:10.3389/fmed.2026.1845645)
Supplement: Supplementary file 1 [file Data_Sheet_1.pdf]

Additional file 1

Perioperative hypoalbuminemia predicts postoperative survival: a large cohort study with global context

Yangqi Chu et al.

eMethod 1: Detailed matching and confounding variables, and outcome definitions................................2

eMethod 2: Detailed statistical analyses...4

Table S1. Data on postoperative mortality and perioperative confounding factors in the preoperative non-hypoalbuminemia and hypoalbuminemia groups before propensity score matching.........................5

Table S2. Data on postoperative mortality and perioperative confounding factors in the postoperative non-hypoalbuminemia and hypoalbuminemia groups before propensity score matching.........................7

Table S3. Cox regression analysis for long-term survival (three-year) and short-term survival (three-month) and hypoalbuminemia after propensity score matching in the complete-case cohort................10

Table S4. Interaction analysis of preoperative/postoperative hypoalbuminemia with age group and sex for 3-month and 3-year mortality after propensity score matching..11

Figure S1-S6. Standardized mean differences (SMDs) distribution figures of perioperative confounding factors before and after propensity score matching...12

Figure S7. Kaplan-Meier survival curves for the matched cohorts comparing patients with and without hypoalbuminemia after propensity score matching..18

eMethod 1: Detailed confounding variables, and outcome definitions

This study was approved by the Ethics Committee of Union Hospital, Tongji Medical College, Huazhong University of Science and Technology. The committee also waived the requirement for written informed consent, as the research utilized only aggregated and non-identifiable patient data. Patient information was retrieved from the electronic clinical records of Wuhan Union Hospital.

1. Detailed matching variables

1) Sex: Male; Female

2) Age: age at the year of surgery; Unit: years

3) BMI: Body Mass Index: Weight (kg) divided by height (m) squared, Unit: kg/m^2

4) ASAPS: American society of Anesthesiologists physical status: I; II; III; IV and higher

5) Previous surgical history: yes; no

6) Previous heart disease: yes; no

7) Previous diabetes: yes; no

8) Previous hypertension: yes; no

9) History of drinking: yes; no

10) History of smoking: yes; no

11) Antihypertensive medication use: yes; no

12) Corticosteroid medication use: yes; no

13) Previous cerebral infarction: yes; no

14) Duration of surgery: Unit: hours

15) Type of surgery: containing ear, nose & throat [ENT] surgery; Gynecological surgery; Gastrointestinal tract surgery; Orthopedic surgery; Urological surgery; General surgery; Nervous system surgery; Thoracic surgery; Ophthalmic surgery

16) Surgery grade: intermediate; major; minor

17) Night shifts: from 18:00~8:00

18) Emergency: yes; no

19) Type of anesthesia: General anesthesia; Intraspinal anesthesia; Local anesthesia; Regional anesthesia; Combined intravenous and inhalation anesthesia

20) Transfusion: yes; no

21) Intraoperative blood transfusion amount: Unit: ml

22) Intraoperative use of vasoactive drugs: yes; no

2. Detailed outcome definitions

In this study, the final endpoint of the hospital data analysis was postoperative mortality derived from the China's National Disease Surveillance Points (DSP) system spanning the years 2014 to 2021. According to the survival time of patients, the death outcome was further divided into short-term death (3 months after operation) and long-term death (3 years after operation).

This DSP system is established and managed by the Chinese Center for Disease Control and Prevention (China CDC). After a significant upgrade in 2013, the DSP system expanded its surveillance points to 605, covering urban (208 points) and rural (397 points) areas across all 31 provinces/autonomous regions/municipalities (hereafter termed provinces) of the Chinese mainland [1]. Mortality data are collected from a wide range of sources, encompassing hospitals, private residences, and various other locations. Trained professionals based at local hospitals or branches of the CDC utilize a standardized protocol to ascertain the causes of death. The DSP system has been validated in prior research as a robust

tool for capturing both national and regional data [2, 3]. To maintain the integrity and consistency of the data, annual training sessions are implemented, focusing on the standardized protocol. These sessions incorporate random audits to verify the precision of disease classification and to identify any duplications. Additionally, retrospective reviews are periodically conducted to assess the extent of underreporting and to ensure the accuracy and completeness of disease coding. Every three years, underreporting surveys are executed to evaluate the DSP system's comprehensiveness and to rectify any potential biases stemming from underreporting [4].

Patients were stratified into four groups based on the presence or absence of hypoalbuminemia before and after surgery. Postoperative serum albumin was measured within 7 days after surgery. The lowest value recorded during this period was used to define postoperative hypoalbuminemia (<35 g/L). Patients without albumin measurements within this time window were excluded from the analysis.

3. Handling of Missing Data

Variables with >10% missingness, including annual household income and educational level, were excluded from the propensity score model.

For variables with ≤10% missingness, including BMI and intraoperative blood transfusion amount, multiple imputation (5 imputations) was performed using the MICE package in R.

Reference

1. Li ML, Qi JL, Ma YQ, et al.: National age-specific mortality trends for cervical and breast cancers in urban-rural areas of China from 2009 to 2021: a population-based analysis. Mil Med Res 2024;11:55.
2. Liu J, Zhang L, Yan Y, et al.: Excess mortality in Wuhan city and other parts of China during the three months of the covid-19 outbreak: findings from nationwide mortality registries. BMJ 2021;372:n415.
3. Zhao ZP, Wang LM, Li YC, et al.: [Provincial representativeness assessment of China Non-communicable and Chronic Disease Risk Factor Surveillance System in 2013]. Zhonghua Yu Fang Yi Xue Za Zhi 2018;52:165-169.
4. Liu S, Wu X, Lopez AD, et al.: An integrated national mortality surveillance system for death registration and mortality surveillance, China. Bull World Health Organ 2016;94:46-57.

eMethod 2: Detailed statistical analyses

1. Sample size calculation

The sample size was determined using the available data from all patients who underwent surgery at Wuhan Union Hospital between January 2014 and December 2018. A statistical power calculation was not conducted prior to the study.

2. Propensity score matching

Matching was performed using 1: 1 matching protocol without replacement, with a caliper of 0.05 SD of the logit of the propensity score. The propensity score matching was conducted in the R environment, version R version 4·2·0 (April, 2022), with “MatchIt” package.

3. Cox regression analyses

After propensity score matching, stratified Cox proportional hazards regression models were used to estimate hazard ratios (HRs) and 95% confidence intervals (CIs) for postoperative mortality associated with preoperative and postoperative hypoalbuminemia.

Table S1. Data on postoperative mortality and perioperative confounding factors in the preoperative non-hypoalbuminemia and the hypoalbuminemia group before propensity score matching

	Preoperative non-hypoalbuminemia (N=170640)	Preoperative hypoalbuminemia (N=29968)	*P*-value
Postoperative death within 3 months	1001 (0.6%)	879 (2.9%)	<0.001
Postoperative death within 3 years	7026 (4.1%)	3403 (11.4%)	<0.001
Age	38.9 (21.0)	46.9 (18.6)	<0.001
Sex			
female	82175 (48.2%)	15615 (52.1%)	<0.001
male	88465 (51.8%)	14353 (47.9%)	
BMI	22.8 (5.00)	23.6 (4.87)	<0.001
ASAPS			
Ⅰ	18916 (11.1%)	1231 (4.1%)	
Ⅱ	63452 (37.2%)	10760 (35.9%)	
Ⅲ	38015 (22.3%)	7591 (25.3%)	<0.001
Ⅳ	50116 (29.4%)	10295 (34.4%)	
Ⅴ	137 (0.1%)	87 (0.3%)	
Previous surgical history	58542 (34.3%)	13074 (43.6%)	<0.001
Previous heart disease	10072 (5.9%)	1479 (4.9%)	<0.001
Previous diabetes	6157 (3.6%)	2217 (7.4%)	<0.001
Previous hypertension	18188 (10.7%)	4095 (13.7%)	<0.001
History of drinking	15116 (8.9%)	3746 (12.5%)	<0.001
History of smoking	18753 (11.0%)	4721 (15.8%)	<0.001

Preoperative antihypertensive medication use	3411 (2.0%)	915 (3.1%)	<0.001
Preoperative corticosteroid medication use	5296 (3.1%)	2241 (7.5%)	<0.001
Previous cerebral infarction	1229 (0.7%)	386 (1.3%)	<0.001
Duration of surgery	1.94 (1.60)	2.41 (1.79)	<0.001
Type of surgery			
ENT surgery	22798 (13.4%)	434 (1.4%)	
Gynecological surgery	10663 (6.2%)	6193 (20.7%)	
Gastrointestinal tract surgery	14988 (8.8%)	5825 (19.4%)	
Orthopedic surgery	29701 (17.4%)	4171 (13.9%)	
Urological surgery	8331 (4.9%)	1315 (4.4%)	
General surgery	52816 (31.0%)	8337 (27.8%)	<0.001
Nervous system surgery	7183 (4.2%)	1044 (3.5%)	
Cardiac surgery	12070 (7.1%)	1304 (4.4%)	
Thoracic surgery	6004 (3.5%)	805 (2.7%)	
Ophthalmic surgery	2328 (1.4%)	90 (0.3%)	
Grade of surgery			
Intermediate	63683 (37.3%)	17504 (58.4%)	
Major	25257 (14.8%)	3153 (10.5%)	<0.001
Minor	81700 (47.9%)	9311 (31.1%)	
Night shifts	16728 (9.8%)	5368 (17.9%)	<0.001
Emergency surgery	38812 (22.7%)	10824 (36.1%)	<0.001
Type of anesthesia			
General anesthesia	148305 (86.9%)	23349 (77.9%)	
Intraspinal anesthesia	14690 (8.6%)	5751 (19.2%)	<0.001
Local anesthesia	1034 (0.6%)	212 (0.7%)	
Regional anesthesia	6611 (3.9%)	656 (2.2%)	

Combined intravenous and inhalation anesthesia	10980 (6.4%)	2314 (7.7%)	<0.001
Transfusion	7594 (4.5%)	3573 (11.9%)	<0.001
Intraoperative blood transfusion amount	31.7 (337)	100 (456)	<0.001
Intraoperative use of vasoactive drugs	16636 (9.7%)	4272 (14.3%)	<0.001

[a]Abbreviation: BMI, Body Mass index; ASAPS, American society of Anesthesiologists physical status; ENT surgery, ear, nose, and throat surgery.

[b]Data are presented as the number (percentage) for categorical variables and as the mean ± SD or median (minimum, maximum) for continuous variables, as appropriate.

[c]The independent samples t test was used to compare continuous variables and the chi-square test to compare categorical variables between groups.

Table S2. Data on postoperative mortality and perioperative confounding factors in the postoperative non-hypoalbuminemia and the hypoalbuminemia group before propensity score matching

	**Postoperative non-hypoalbuminemia (N=85196)**	**Postoperative hypoalbuminemia (N=115412)**	***P*-value**
Postoperative death within 3 months	221 (0.3%)	1659 (1.4%)	<0.001
Postoperative death within 3 years	2051 (2.4%)	8378 (7.3%)	<0.001
Age	34.1 (21.4)	44.5 (19.3)	<0.001
Sex			
female	40355 (47.4%)	57435 (49.8%)	<0.001
male	44841 (52.6%)	57977 (50.2%)	
BMI	22.4 (5.24)	23.3 (4.76)	<0.001
ASAPS			
Ⅰ	11044 (13.0%)	9103 (7.9%)	<0.001
Ⅱ	32000 (37.6%)	42212 (36.6%)	
Ⅲ	18462 (21.7%)	27144 (23.5%)	
Ⅳ	23643 (27.8%)	36768 (31.9%)	

V	47 (0.1%)	177 (0.2%)	
Previous surgical history	28585 (33.6%)	43031 (37.3%)	<0.001
Previous heart disease	5746 (6.7%)	5805 (5.0%)	<0.001
Previous diabetes	2428 (2.8%)	5946 (5.2%)	<0.001
Previous hypertension	7314 (8.6%)	14969 (13.0%)	<0.001
History of drinking	6756 (7.9%)	12106 (10.5%)	<0.001
History of smoking	7882 (9.3%)	15592 (13.5%)	<0.001
Preoperative antihypertensive medication use	1214 (1.4%)	3112 (2.7%)	<0.001
Preoperative corticosteroid medication use	2447 (2.9%)	5090 (4.4%)	<0.001
Previous cerebral infarction	395 (0.5%)	1220 (1.1%)	<0.001
Duration of surgery	1.69 (1.37)	2.24 (1.77)	<0.001
Type of surgery			
ENT surgery	11615 (13.6%)	11617 (10.1%)	
Gynecological surgery	4040 (4.7%)	12816 (11.1%)	
Gastrointestinal tract surgery	6197 (7.3%)	14616 (12.7%)	
Orthopedic surgery	15788 (18.5%)	18084 (15.7%)	
Urological surgery	3863 (4.5%)	5783 (5.0%)	
General surgery	28761 (33.8%)	32392 (28.1%)	<0.001
Nervous system surgery	2775 (3.3%)	5452 (4.7%)	
Cardiac surgery	6527 (7.7%)	6847 (5.9%)	
Thoracic surgery	2529 (3.0%)	4280 (3.7%)	
Ophthalmic surgery	1264 (1.5%)	1154 (1.0%)	
Grade of surgery			
Intermediate	29888 (35.1%)	51299 (44.4%)	
Major	11831 (13.9%)	16579 (14.4%)	<0.001
Minor	43477 (51.0%)	47534 (41.2%)	

Night shifts	8471 (9.9%)	13625 (11.8%)	<0.001
Emergency surgery	19362 (22.7%)	30274 (26.2%)	<0.001
Type of anesthesia			
General anesthesia	74526 (87.5%)	97128 (84.2%)	<0.001
Intraspinal anesthesia	6912 (8.1%)	13529 (11.7%)	
Local anesthesia	523 (0.6%)	723 (0.6%)	
Regional anesthesia	3235 (3.8%)	4032 (3.5%)	
Combined intravenous and inhalation anesthesia	6161 (7.2%)	7133 (6.2%)	<0.001
Transfusion	2569 (3.0%)	8598 (7.4%)	<0.001
Intraoperative blood transfusion amount	17.5 (244)	60.0 (422)	<0.001
Intraoperative use of vasoactive drugs	7206 (8.5%)	13702 (11.9%)	<0.001

[a]Abbreviation: BMI, Body Mass index; ASAPS, American society of Anesthesiologists physical status; ENT surgery, ear, nose, and throat surgery.

[b]Data are presented as the number (percentage) for categorical variables and as the mean ± SD or median (minimum, maximum) for continuous variables, as appropriate.

[c]The independent samples t test was used to compare continuous variables and the chi-square test to compare categorical variables between groups.

Table S3. Cox Regression Analysis for long-term survival (three-year) and short-term survival (three-month) and hypoalbuminemia after propensity score matching in the complete-case cohort (excluding any patient with missing covariates)

	Long-term survival (three-year)			Short-term survival (three-month)		
	**Hazard Ratio**	**95% CI**	***P*-value**	**Hazard Ratio**	**95% CI**	***P*-value**
Preoperative hypoalbuminemia	1.601	1.514, 1.693	**<0.001**	2.319	2.047, 2.628	**<0.001**
Postoperative hypoalbuminemia	1.894	1.785, 2.011	**<0.001**	3.165	2.684, 3.733	**<0.001**

Table S4. Interaction analysis of preoperative/postoperative hypoalbuminemia with age group and sex for 3-month and 3-year mortality after propensity score matching

	Long-term survival (three-year)			Short-term survival (three-month)		
	**Hazard Ratio**	**95% CI**	***P for interaction (vs reference)***	**Hazard Ratio**	**95% CI**	***P for interaction (vs reference)***
Preoperative hypoalbuminemia						
Sex						
Male cohort	3.213	3.053, 3.382	0.056	5.650	5.048, 6.324	0.353
Female	2.592	2.421, 2.774	Reference	4.440	3.812, 5.170	Reference
Age						
Age>70	1.402	1.242, 1.583	**<0.001**	1.926	1.469, 2.524	**0.004**
Age<5	0.499	0.150, 1.658	0.182	0.667	0.111, 3.991	0.067
Age between 5 and 70 years	1.782	1.673, 1.897	Reference	2.683	2.324, 3.096	Reference
Postoperative hypoalbuminemia						
Sex						
Male cohort	3.236	3.047, 3.437	**0.005**	6.787	5.640, 8.167	**0.043**
Female	2.992	2.760, 3.243	Reference	4.195	3.379, 5.207	Reference
Age						
Age>70	1.391	1.170, 1.653	**<0.001**	2.594	1.567, 4.294	0.617
Age<5	2.755	1.227, 6.189	0.194	7.510	1.717, 32.840	0.137
Age between 5 and 70 years	1.993	1.883, 2.111	Reference	3.297	2.792, 3.894	Reference

Figure S1. Standardized Mean Differences of Perioperative Confounding Factors in Preoperative and Postoperative Hypoalbuminemia Groups Before and After Propensity Score Matching (Age > 70 Years). BMI=Body Mass index; ASAPS=American society of Anesthesiologists physical status; ENT surgery=ear, nose, and throat surgery.

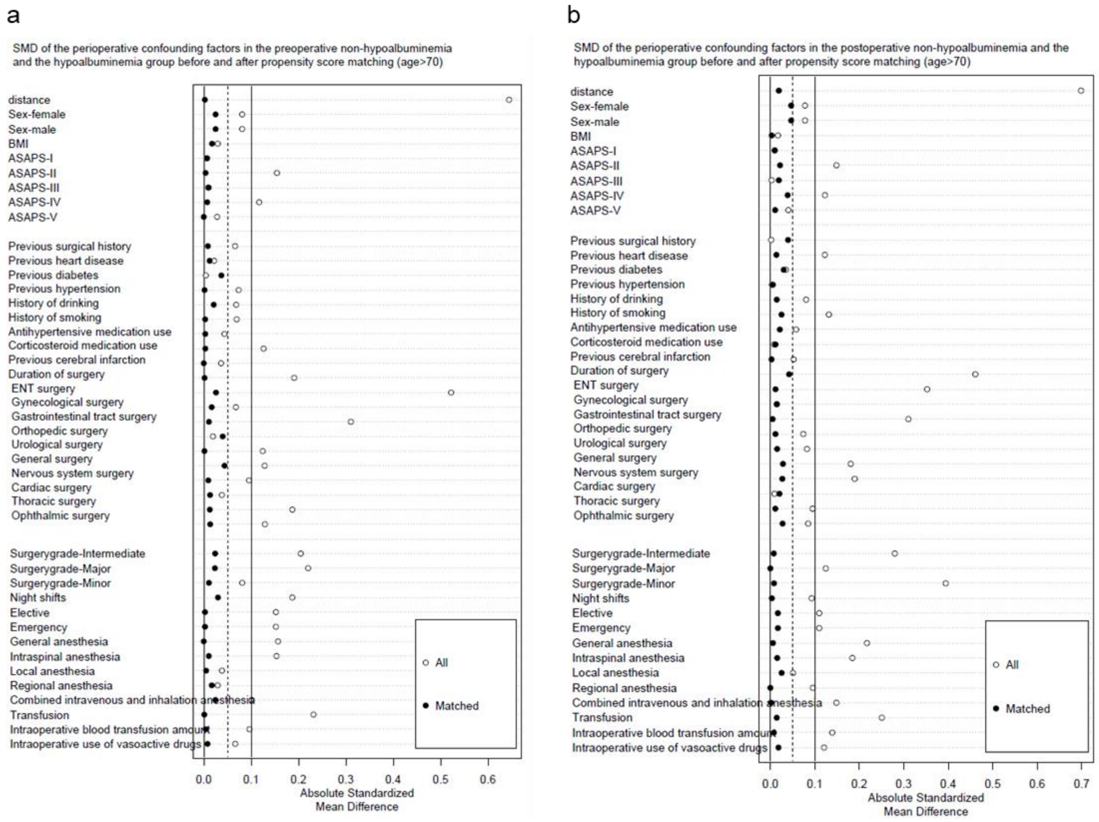

Figure S2. Standardized Mean Differences of Perioperative Confounding Factors in Preoperative and Postoperative Hypoalbuminemia Groups Before and After Propensity Score Matching (Age < 5 Years). BMI=Body Mass index; ASAPS=American society of Anesthesiologists physical status; ENT surgery=ear, nose, and throat surgery.

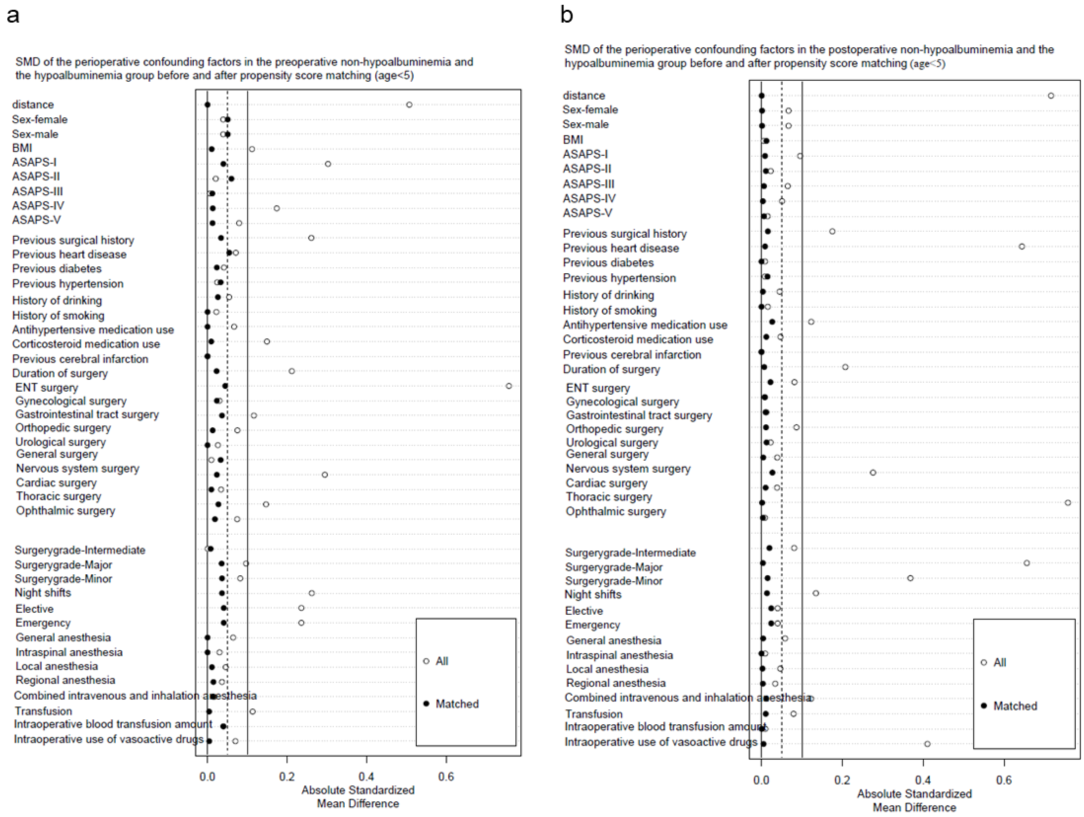

Figure S3. Standardized Mean Differences of Perioperative Confounding Factors in Preoperative and Postoperative Hypoalbuminemia Groups Before and After Propensity Score Matching (Age Between 5 and 70 Years). BMI=Body Mass index; ASAPS=American society of Anesthesiologists physical status; ENT surgery=ear, nose, and throat surgery.

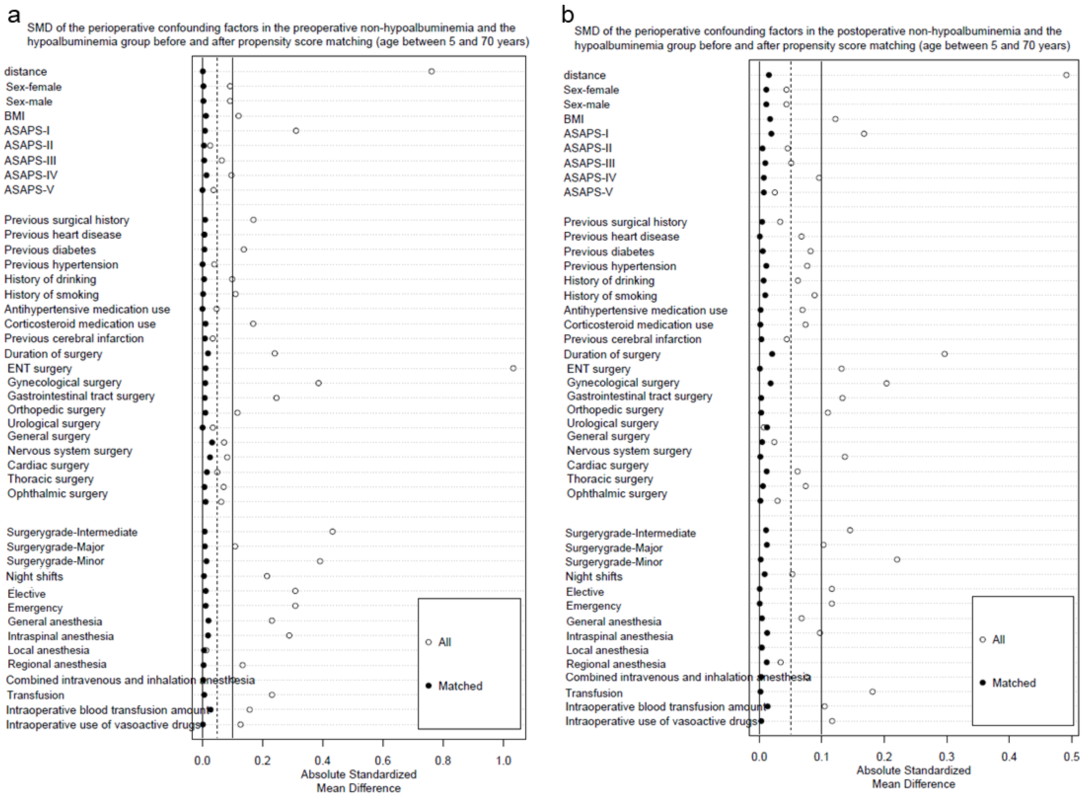

Figure S4. Standardized Mean Differences of Perioperative Confounding Factors in Preoperative and Postoperative Hypoalbuminemia Groups Before and After Propensity Score Matching (Male). BMI=Body Mass index; ASAPS=American society of Anesthesiologists physical status; ENT surgery=ear, nose, and throat surgery.

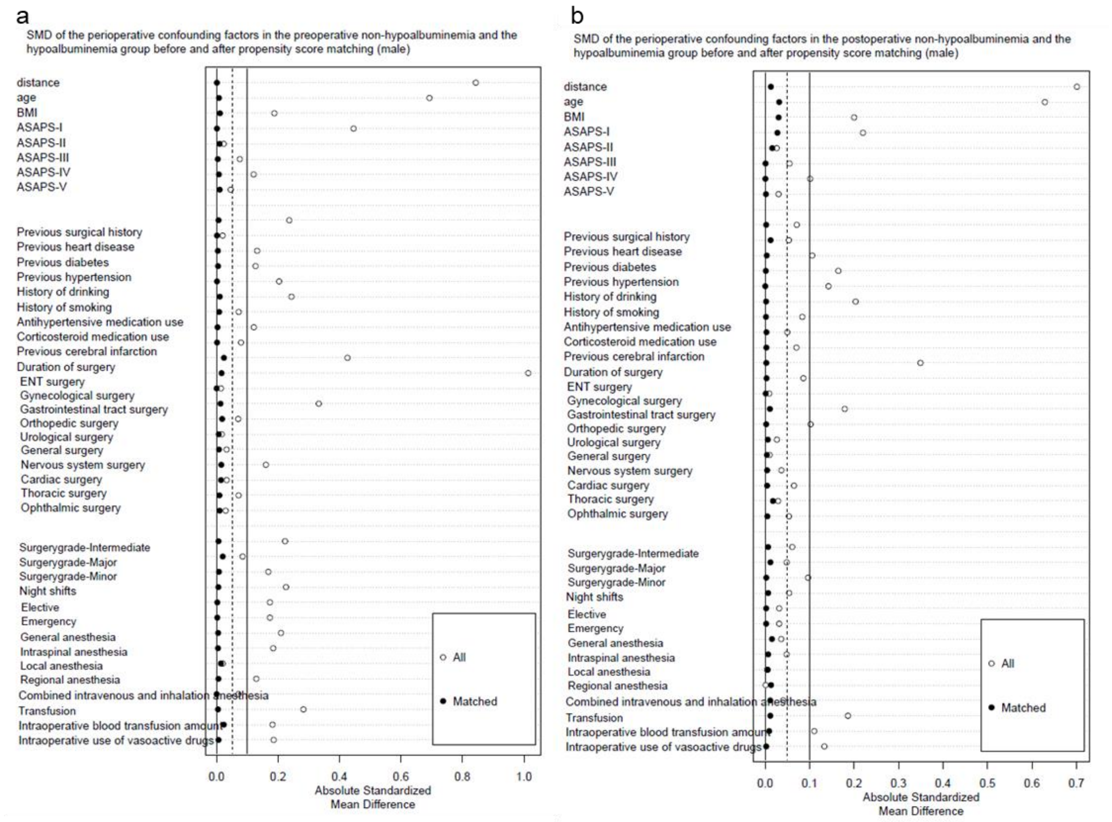

Figure S5. Standardized Mean Differences of Perioperative Confounding Factors in Preoperative and Postoperative Hypoalbuminemia Groups Before and After Propensity Score Matching (Female). BMI=Body Mass index; ASAPS=American society of Anesthesiologists physical status; ENT surgery=ear, nose, and throat surgery.

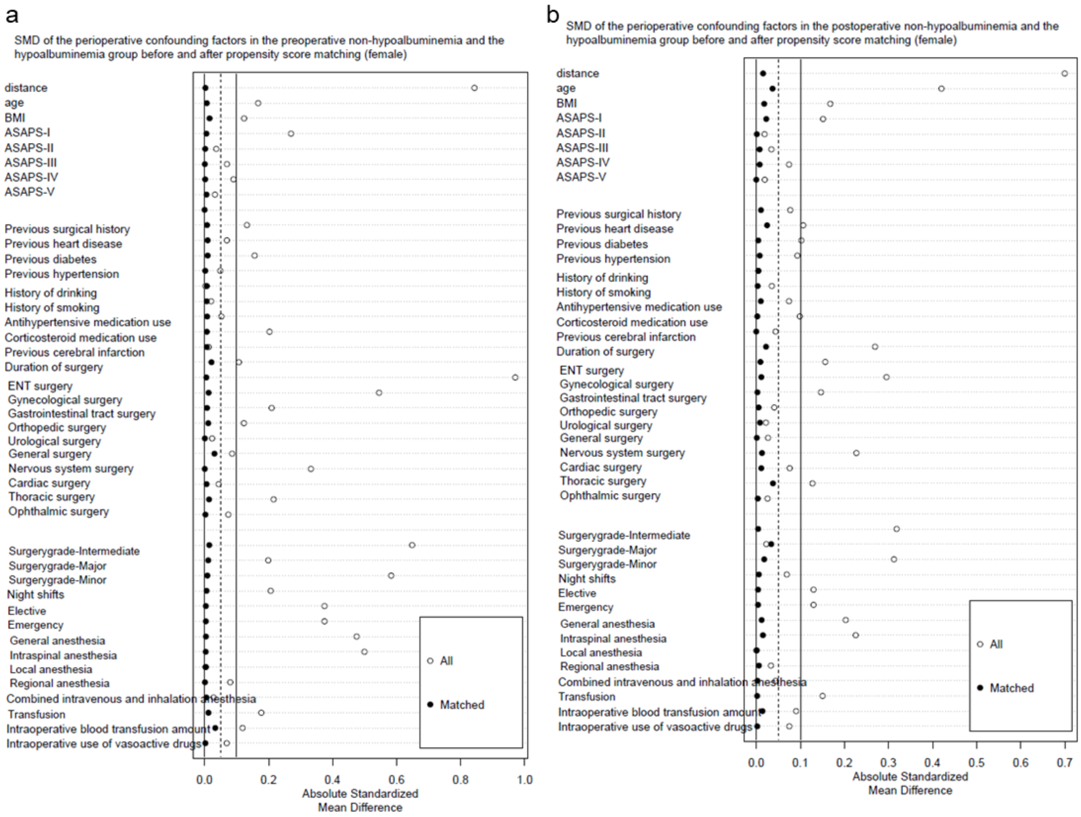

Figure S6. Standardized Mean Differences of Perioperative Confounding Factors in the Entire Cohort Before and After Propensity Score Matching for Preoperative and Postoperative Hypoalbuminemia. BMI=Body Mass index; ASAPS=American society of Anesthesiologists physical status; ENT surgery=ear, nose, and throat surgery.

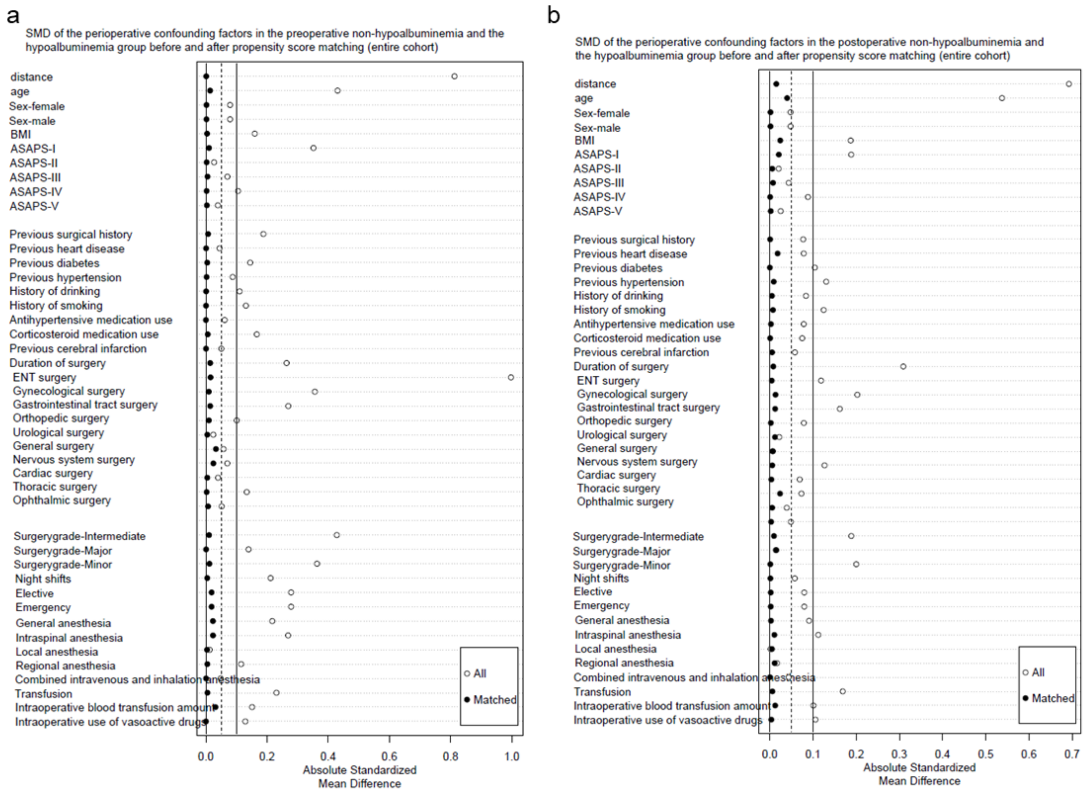

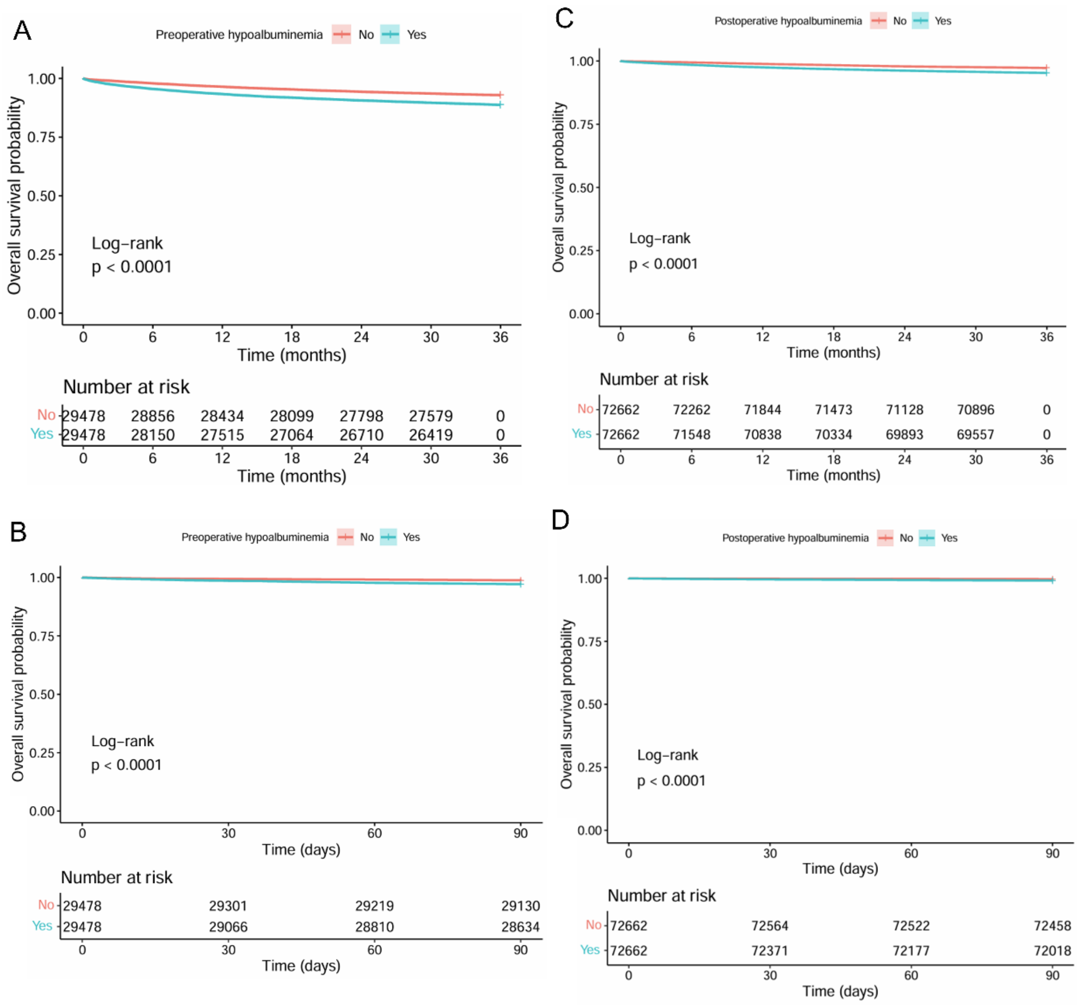

Figure S7. Kaplan-Meier survival curves for the matched cohorts comparing patients with and without hypoalbuminemia after propensity score matching.

(A) Preoperative hypoalbuminemia – 3-year survival (36 months).

(B) Preoperative hypoalbuminemia – 3-month survival.

(C) Postoperative hypoalbuminemia – 3-year survival.

(D) Postoperative hypoalbuminemia – 3-month survival.

Log-rank p values are shown for each comparison. Risk tables are displayed below each curve.
